# Regulation of Cardiac Fibroblast GLS1 Expression by Scleraxis

**DOI:** 10.3390/cells11091471

**Published:** 2022-04-27

**Authors:** Sikta Chattopadhyaya, Raghu S. Nagalingam, D. Allison Ledingham, Teri L. Moffatt, Danah S. Al-Hattab, Pavit Narhan, Matthew T. Stecy, Kimberley A. O’Hara, Michael P. Czubryt

**Affiliations:** 1 Institute of Cardiovascular Sciences, St. Boniface Hospital Albrechtsen Research Centre, Winnipeg, MB R2H 2A6, Canada; schattopadhyaya@sbrc.ca (S.C.); raghu.n.sundaresan@gmail.com (R.S.N.); aledingham@sbrc.ca (D.A.L.); tmoffatt@sbrc.ca (T.L.M.); dalhattab@sbrc.ca (D.S.A.-H.); pavitnarhan@gmail.com (P.N.); stecym@myumanitoba.ca (M.T.S.); kohara@sbrc.ca (K.A.O.); 2 Department of Physiology and Pathophysiology, Rady Faculty of Health Sciences, University of Manitoba, Winnipeg, MB R3E 0J9, Canada

**Keywords:** cardiac fibrosis, energy metabolism, fibroblast, myofibroblast, glutaminolysis, transcription, gene regulation

## Abstract

Fibrosis is an energy-intensive process requiring the activation of fibroblasts to myofibroblasts, resulting in the increased synthesis of extracellular matrix proteins. Little is known about the transcriptional control of energy metabolism in cardiac fibroblast activation, but glutaminolysis has been implicated in liver and lung fibrosis. Here we explored how pro-fibrotic TGFβ and its effector scleraxis, which drive cardiac fibroblast activation, regulate genes involved in glutaminolysis, particularly the rate-limiting enzyme glutaminase (GLS1). The GLS1 inhibitor CB-839 attenuated TGFβ-induced fibroblast activation. Cardiac fibroblast activation to myofibroblasts by scleraxis overexpression increased glutaminolysis gene expression, including GLS1, while cardiac fibroblasts from scleraxis-null mice showed reduced expression. TGFβ induced GLS1 expression and increased intracellular glutamine and glutamate levels, indicative of increased glutaminolysis, but in scleraxis knockout cells, these measures were attenuated, and the response to TGFβ was lost. The knockdown of scleraxis in activated cardiac fibroblasts reduced GLS1 expression by 75%. Scleraxis transactivated the human GLS1 promoter in luciferase reporter assays, and this effect was dependent on a key scleraxis-binding E-box motif. These results implicate scleraxis-mediated GLS1 expression as a key regulator of glutaminolysis in cardiac fibroblast activation, and blocking scleraxis in this process may provide a means of starving fibroblasts of the energy required for fibrosis.

## 1. Introduction

Cardiac fibrosis may be considered an aberrant wound healing process. In non-cardiac tissues, the tissue injury response normally results in the synthesis of extracellular matrix (ECM) proteins balanced by the degradation of existing ECM proteins by matrix metalloproteinases (MMPs); this typically restores tissue function without causing fibrosis [1]. In the myocardium, however, pathological stresses such as hypertension, valve disease, diabetes, or myocardial infarction result in a prolonged and/or dysregulated wound healing response that leads to scar formation or interstitial fibrosis, which interferes with cardiac function in a manner that frequently leads to heart failure or death [1,2]. Resident fibroblasts are primarily responsible for the synthesis of ECM components including collagens and proteoglycans; in response to pro-fibrotic stimuli, including growth factors, cytokines, and mechanical stretch, fibroblasts become activated to myofibroblasts that sharply upregulate ECM production [1,3,4,5,6]. While cardiac fibrosis is now recognized as a major contributor to morbidity and mortality, there remain no therapeutic strategies for its management [7].

The synthesis of ECM components, including large macromolecules such as fibrillar collagens, is an energy-intensive process. Metabolic reprogramming, including mitochondrial biogenesis, is required for the activation of human fetal lung fibroblasts to myofibroblasts [8]. The underlying mechanisms governing energy metabolism in fibroblasts and myofibroblasts, however, remain poorly defined, and the metabolic processes themselves appear to vary across tissues. Glycolysis is increased in lung fibroblasts during activation to myofibroblasts [9]. Conversely, the promotion of fatty acid oxidation attenuates tubulointerstitial fibrosis; thus, energy requirements in fibrosis likely require specific alterations in metabolism [10]. Glutaminolysis utilizes glutamine metabolism for energy production via the conversion of glutamine to glutamate and ammonia by the rate-limiting enzyme glutaminase (GLS), and the subsequent conversion of glutamate to α-ketoglutarate (α-KG) by glutamate dehydrogenase (GDH), glutamate oxaloacetate transaminase (GOT), or the glutamate pyruvate transaminase (GPT). α-KG then enters the TCA cycle to meet energy demands [11,12]. Glutaminolysis has already been identified as a major target for the treatment of liver and pulmonary fibrosis [13,14]. A recent study in mouse embryonic fibroblasts identified increases in glycolysis and glutaminolysis as necessary to induce fibroblast activation and conversion to myofibroblasts, with α-KG-dependent histone demethylases in turn activating myofibroblast genes [15]. The role of glutaminolysis specifically in cardiac fibroblasts remains unclear.

The transcription factor scleraxis plays a key role in the activation of fibroblasts to myofibroblasts via direct transcriptional activation of key fibrotic genes, leading to increased ECM synthesis [5]. TGFβ is a potent inducer of fibrosis in many tissues, and it was recently shown that the activation of human lung fibroblasts to myofibroblasts required glutaminolysis, with GLS1 expression dependent on TGFβ and its downstream effector SMAD3 [16]. Since we previously showed that TGFβ-mediated activation of cardiac fibroblasts and the induction of fibrotic gene expression require scleraxis, we examined whether scleraxis may play a role in cardiac fibroblast glutaminolysis [5,17,18].

Here we report that, similar to lung fibroblasts, cardiac fibroblasts upregulated GLS1 expression in response to TGFβ, and that the GLS1 inhibitor CB-839 attenuated fibroblast activation. Scleraxis over-expression in cardiac fibroblasts induced the expression of multiple enzymes in the glutaminolysis pathway, including GLS1, GLS2, and GOT2. Conversely, GLS1 and GDH1 expression was decreased in scleraxis knockout cardiac fibroblasts. Scleraxis knockdown attenuated GLS1 expression and dramatically blunted GLS1 upregulation by TGFβ. Cellular concentrations of glutamate and glutamine increased with TGFβ treatment, indicative of increased throughput in the glutaminolysis pathway, but this effect was attenuated by scleraxis knockout. Scleraxis regulated GLS1 gene expression via a specific E-box binding site in the human GLS1 promoter. Scleraxis thus appears to be a key regulator of glutaminolysis in cardiac fibroblast activation via the transactivation of the GLS1 gene.

## 2. Materials and Methods

### 2.1. Cardiac Fibroblast Isolation and Culture

Primary rat cardiac fibroblasts (RCFs) from adult male Sprague Dawley rat hearts were isolated by enzymatic digestion as previously described [19,20]. Primary mouse cardiac fibroblasts (MCFs) were isolated using enzymatic digestion from scleraxis germline knockout (KO), or C57BL6 wild-type (WT) control mice as previously described [5]. Primary adult RCFs and MCFs were maintained in Dulbecco’s Modified Eagle’s Medium (DMEM)/F12 medium supplemented with 10% fetal bovine serum and 1% penicillin-streptomycin. NIH3T3 mouse fibroblasts were cultured in DMEM containing 10% fetal bovine serum and 1% penicillin-streptomycin. Adult human cardiac myofibroblasts (Cell Applications, San Diego, CA, USA) were cultured in DMEM/F12 medium supplemented with 20% fetal bovine serum and 1% penicillin-streptomycin. Primary rat/mouse cardiac fibroblasts harvested within 6 h of initial plating were used as zero passage (P0) cells or were passaged once at 48 h after initial plating and used as activated fibroblasts (P1) or were passaged a second time at 96 h after initial plating and used as myofibroblasts (P2) as described previously [5]. All cells were maintained at 37 °C and 5% CO_2_. For TGFβ_1_ treatment, P1 cells were plated on either 35 mm or 6-well cell culture dishes and maintained in 10% serum-containing medium for 16 h to reach 70% confluence, then serum starved for 6 h prior to treatment with TGFβ_1_ (10 ng/mL) or vehicle (PBS). After 24 h treatment, cells were harvested for processing. For inhibition of glutaminolysis, cells were grown to 70% confluence, serum-starved for 24 h, then treated with the GLS1 inhibitor CB-839 (0.3 μM) or vehicle (DMSO) followed one hour later by TGFβ_1_ or vehicle treatment for 24 h, then cells were harvested and processed.

For scleraxis adenoviral overexpression experiments, RCFs at P1 were grown within 16 h to reach 70% confluence and transfected with adenoviruses encoding either scleraxis (AdScx) or Green Fluorescent Protein (AdGFP) at a multiplicity of infection of 100 in serum-free medium for 48 h. For scleraxis knockdown experiments, RCFs at P1 were grown for 16 h to reach 70% confluence, then serum starved for 6 h and transfected with scleraxis knockdown adenovirus (AdshScx) or control virus (AdshLacZ) at a multiplicity of infection of 100 in serum-free medium for 72 h, with or without TGFβ_1_ (10 ng/mL) added at 48 h after adenovirus addition. For luciferase assays, NIH3T3 fibroblasts were grown in 6-well plates to reach 60–70% confluence and maintained in low serum (0.5%) for 2 h prior to transfection. For chromatin immunoprecipitation assays, adult human cardiac myofibroblasts were grown in 150 mm dishes for 16 h to reach 70–80% confluence, then serum starved for 30 h prior to harvest for further analysis. For intracellular glutamine/glutamate assay, freshly isolated wild type or scleraxis knockout MCFs were plated on 12-well dishes to reach 60–70% confluence, followed by serum starvation for 24 h prior to treatment with TGFβ_1_ (10 ng/mL) or vehicle.

### 2.2. Quantitative Real-Time PCR

Total RNA from primary adult rat or mouse cardiac fibroblasts were harvested using a Monarch Total RNA Miniprep Kit (New England Biolabs, Whitby, ON, Canada) according to the manufacturer’s instructions. cDNA was generated using an iScript cDNA Synthesis kit (Bio-Rad, Mississauga, ON, Canada), and SsoAdvanced Universal SYBR Green Supermix (Bio-Rad) was used for qPCR amplification according to the manufacturer’s instructions. PCR amplification was performed in a CFX384 Touch Real-Time PCR (Bio-Rad, Canada). The cycling conditions were 95 °C (3 min), followed by 40 cycles of denaturation at 95 °C (15 s) and extension at 62 °C (30 s). After amplification, a continuous melt curve was generated from 60 to 95 °C to confirm the amplification of single amplicons. Relative gene expression was calculated using the 2^−ΔΔCt^ method with normalization to Gapdh. Primer sequences are listed in Table S1.

### 2.3. Western Blotting

For whole-cell lysates, 30 μg protein samples were resolved on 10% SDS-PAGE gels and transferred to PVDF membranes, which were then blocked with 5% nonfat milk in Tris-Buffered Saline with 0.1% Tween-20 (TBS-T) for 1 h at room temperature. For detection of proteins of interest, the membranes were incubated with primary antibodies for GLS1 (1:1000; PA5-35365; ThermoFisher Scientific, Ottawa, ON, Canada), ALDOC (1:1000; PA5-27659; ThermoFisher Scientific, Canada), or Acot2 (1:1000; 15633-1-AP; ThermoFisher Scientific, Canada) in 2% nonfat milk in TBS-T at 4 °C overnight, followed by washing and incubation with secondary horseradish peroxidase-conjugated goat anti-rabbit antibody (1:10,000; 111-035-003; Jackson Laboratories, Bar Harbor, ME, USA) in TBS-T with 1% nonfat milk for 1 h at room temperature. Protein bands were visualized using Pierce ECL Western blotting chemiluminescent substrate (ThermoFisher Scientific, Canada) and exposure to CL-XPosure film (Mandel Scientific, Guelph, ON, Canada). Protein quantification was conducted using Image J (NIH, Bethesda, MD, USA), with normalization to horseradish peroxidase-conjugated β-actin antibody (1:65,000; SC-47778; Santa Cruz Biotechnology Inc., Dallas, TX, USA). Secreted periostin was similarly assessed using culture medium collected from plated cells, with proteins concentrated using Amicon Ultra-15 centrifugal filters (Millipore Sigma, Oakville, ON, Canada) and resolved on 4–15% TGX Stain-Free Precast Gels (Bio-Rad, Canada). Proteins were transferred to PVDF membranes and probed with primary antibodies for periostin (1:1000; ab14041; Abcam, Toronto, ON, Canada), with normalization to total protein given the lack of a specific loading control for secreted proteins.

### 2.4. Plasmid Constructs and Mutagenesis

The proximal human GLS1 promoter (−1138 to +190 relative to the transcription start site, 1329 base pairs total length) was cloned by PCR amplification from the HPRM39644 pEZX-PG02.1 vector (GeneCopoeia, Rockville, MD, USA) using the primers listed in Table S2, and incorporating restriction sites for SacI (forward primer) and EcoRV (reverse primer). The promoter was then subcloned into the luciferase reporter vector pGL4.10-luc2 (Promega, Madison, WI, USA), generating the construct pGL4.10-hGLS1.

Site-directed mutagenesis of three putative E-box sites was performed sequentially using a QuikChange II XL kit (Agilent Technologies, Santa Clara, CA, USA) and primers listed in Table S3. All constructs were confirmed by sequencing.

### 2.5. Luciferase Assay

NIH3T3 fibroblasts were co-transfected with scleraxis expression vector pcDNA-Scx or empty control vector pcDNA (pcDNA6, ThermoFisher Scientific, Canada), and pGL4.10-hGLS1 with or without E-box mutants using Lipofectamine 3000 (ThermoFisher Scientific, Canada) for 24 h. Renilla luciferase vector pRL was co-transfected as an internal transfection control. Luciferase activity was measured using the Dual Luciferase Reporter Assay System kit (Promega, USA) and a GloMax-Multi+ Multimode Plate Reader (Promega, USA) according to manufacturer’s directions.

### 2.6. Glutamine/Glutamate Assay

Mouse cardiac fibroblasts were harvested after treatment with TGFβ_1_, and the cell lysates were processed as per the manufacturer’s instructions (Glutamine/Glutamate-Glo™ Assay; J8021; Promega, USA) for determining the intracellular glutamine and glutamate concentrations. Two reactions were performed in duplicate per sample in an opaque, white 96-well plate (Corning Costar; 3912; Millipore-Sigma, Canada). For one reaction, the cell lysates were incubated with glutaminase enzyme to provide total glutamine + glutamate concentration; for the second reaction, the cell lysates were incubated without glutaminase enzyme, thereby providing the glutamate concentration alone. Subtracting these two values yielded the glutamine concentration. Luminescence was recorded using a GloMax-Multi+ Multimode Plate Reader (Promega, USA).

### 2.7. Chromatin Immunoprecipitation Assay

Adult human cardiac myofibroblasts were serum starved for 30 h, then cross-linked with 4% paraformaldehyde, and chromatin was sheared into 300–500 base pair fragments by sonication. After sonication, cell lysates were processed using an EZ-Magna ChIP HiSens Chromatin Immunoprecipitation Kit (Millipore-Sigma, Canada), and the experiment was carried out according to the manufacturer’s instructions. For the chromatin pull-down, either anti-scleraxis antibody or non-specific IgG was used [20]. Specific primers were used to amplify the region of the scleraxis-bound E-boxes (E1/E2/E3) in the proximal human GLS1 gene promoter, with assessment by qPCR (Table S4).

### 2.8. Phalloidin Staining

Freshly isolated adult rat cardiac fibroblasts were plated on acid-washed 18 mm glass coverslips in 12-well plates, then treated with CB-839 and/or TGFβ_1_ as above. Cells were fixed with 4% paraformaldehyde for 30 min at room temperature followed by 3 washes in PBS, then permeabilized in 0.1% Triton X-100 in PBS for 5 min followed by 3 washes in PBS. Coverslips were incubated for 1 h at room temperature with CytoPainter Phalloidin-iFluor 594 Reagent (ab176757; Abcam, Canada) as per the manufacturer’s instructions. Staining solution was removed, and coverslips were washed 3 times with PBS. Coverslips were mounted onto slides using ProLong Gold Antifade Mountant with DAPI (ThermoFisher Scientific, Canada). Cells were imaged on a Nikon Eclipse TE2000S fluorescent microscope using a 20× objective. NIS software (Nikon, Melville, NY, USA) was used for image capture, and Image J (NIH, USA) was used to merge captured images.

### 2.9. CCK8 Cell Proliferation Assay

Cell proliferation was analyzed using a Cell Counting Kit-8 (CCK-8) (CK04-011; Dojindo Laboratories, Rockville, MD, USA), according to the manufacturer’s directions. Briefly, rat cardiac fibroblasts were plated in 96-well plates and treated with or without TGFβ_1_ and/or CB-839 as above for 24, 48, or 72 h as above. CCK-8 solution was added to each well, except for 3 baseline control wells from each of the treated groups. Plates were incubated at 37 °C for 1 h, then optical density at 450 nm was measured for each well using a microplate reader, baseline mean was subtracted from each sample value, and results were then normalized to vehicle-treated cells.

### 2.10. Statistical Analysis

Data are reported as mean ± standard deviation for a minimum of three independent biological replicates. To reduce variability, all cells, including control and treatment groups, were isolated and cultured on the same day. Results were analyzed by a two-tailed Student’s t-test or a one-way or two-way analysis of variance (ANOVA), with Tukey post hoc analysis as appropriate, using GraphPad Prism v.9.3.1 (GraphPad Software, San Diego, CA, USA) with *p* < 0.05 considered to be statistically significant. Normality of distribution was confirmed using the Shapiro–Wilk test; non-normally distributed data were analyzed by the Kruskal–Wallis test, followed by Dunn’s multiple comparisons test.

## 3. Results

### 3.1. Interplay of TGFβ and Glutaminolysis in Cardiac Fibroblasts

**Figure 1:**
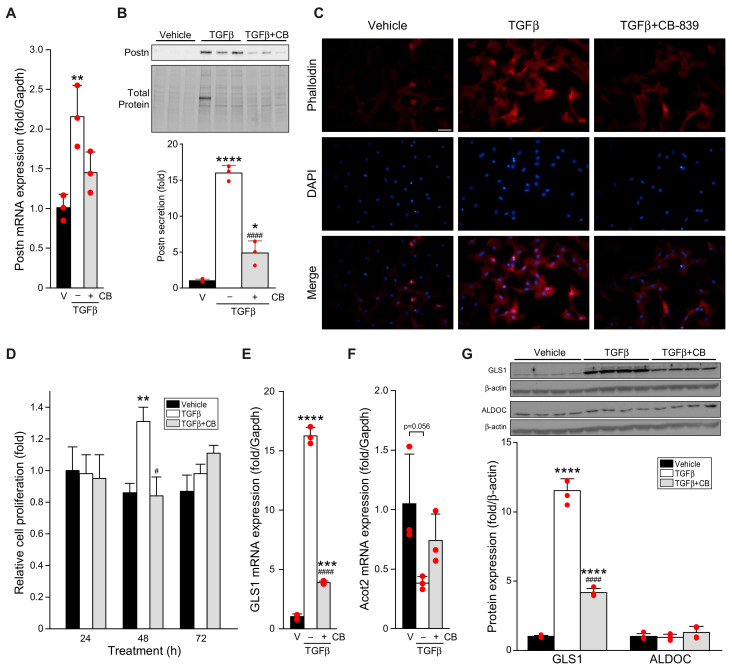
TGFβ-induced fibroblast activation and GLS1 expression is attenuated by CB-839. (**A**–**F**) Rat cardiac fibroblasts were treated for 24 h with vehicle (V) or 10 ng/mL TGFβ_1_, with or without GLS1 inhibitor CB-839 (CB, 0.3 μM), then assayed for expression of periostin (Postn) mRNA by qPCR (**A**), secretion of Postn (**B**), development of stress fibers (**C**), proliferation (**D**), and expression of GLS1 (**E**) and Acot2 mRNA by qPCR (**F**). In a similar experiment, protein expression of GLS1 and aldolase C (ALDOC) was assessed by Western blot (**G**). Statistical significance was determined by one-way ANOVA with the Tukey post hoc test (n = 3–4) (**A**,**B**,**E**–**G**), or by the Kruskal–Wallis test followed by Dunn’s multiple comparisons test (**D**, n = 16–18; ALDOC in **G**, n = 4); images in (**C**) are representative of four independent experiments. * *p* < 0.05, ** *p* < 0.01, *** *p* < 0.001, **** *p* < 0.0001 versus vehicle; # *p* < 0.05, #### *p* < 0.0001 versus TGFβ. Scale bar in (**C**), 50 μm.

TGFβ induces glutaminolysis in lung fibroblasts [16]. We thus examined the interplay of TGFβ and the induction of glutaminolysis gene expression in rat cardiac P1 fibroblasts. TGFβ induced the conversion of fibroblasts to myofibroblasts as assessed by the upregulation and secretion of the myofibroblast marker periostin (Figure 1A,B) [21,22]. TGFβ also increased stress fiber formation indicative of activation to myofibroblasts (Figure 1C) and caused a transient increase in fibroblast proliferation (Figure 1D). These increases occurred concomitantly with an increase in GLS1 expression and a trend towards a decrease in expression of the key fatty acid oxidation gene Acot2 (Figure 1E,F), suggestive of a shift in fuel usage as fibroblasts become myofibroblasts [23].

CB-839 is a well-characterized inhibitor of GLS1 and glutaminolysis that has been shown to attenuate pulmonary fibrosis induced by either bleomycin or TGFβ [14]. The treatment of cardiac fibroblasts with CB-839 attenuated periostin expression and secretion, reduced stress fiber formation, and blocked the increase in proliferation induced by TGFβ, indicative of reduced conversion of fibroblasts to myofibroblasts, while reducing GLS1 expression and attenuating the decrease in Acot2 (Figure 1A–F). Similar results were observed for GLS1 protein expression (Figure 1G). Neither TGFβ nor CB-839 impacted the expression of the glycolysis enzyme aldolase C (ALDOC; Figure 1G).

### 3.2. Scleraxis Upregulates the Key Enzymes of the Glutaminolysis Pathway in Cardiac Fibroblasts

**Figure 2:**
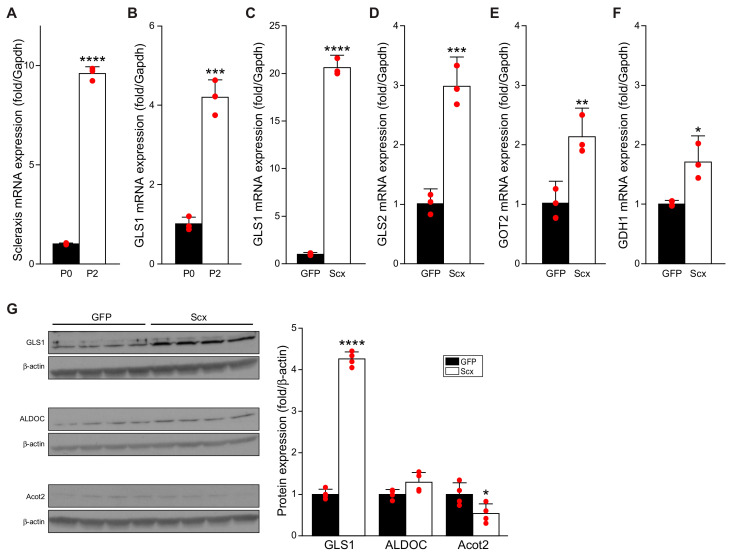
Fibroblast activation and scleraxis overexpression upregulate glutaminolysis gene expression. (**A**,**B**) Non-passaged (P0), freshly-isolated rat cardiac fibroblasts, or twice-passaged P2 rat cardiac myofibroblasts were assayed for mRNA expression of scleraxis (**A**) and GLS1 (**B**) by qPCR. (**C**–**G**) P1 rat cardiac fibroblasts were transfected by adenovirus encoding green fluorescent protein (GFP), or scleraxis (Scx) for 48 h, then assayed by qPCR for expression of GLS1 (**C**), GLS2 (**D**), GOT2 (**E**), and GDH1 (**F**), or were analyzed by Western blot for expression of GLS1, ALDOC, and Acot2 (**G**). Statistical analysis was by the two-tailed Student’s t-test (n = 3–4). * *p* < 0.05, ** *p* < 0.01, *** *p* < 0.001, **** *p* < 0.0001 versus P0 or GFP.

We demonstrated that TGFβ requires scleraxis to mediate the downstream regulation of key target genes such as Col1α2, fibronectin, and α-SMA; that scleraxis alone is sufficient to induce the conversion of cardiac fibroblasts to myofibroblasts; and that scleraxis is upregulated in cardiac myofibroblasts compared to fibroblasts [5,17,18]. We confirmed that scleraxis is upregulated in twice-passaged P2 cardiac myofibroblasts compared to freshly isolated P0 cardiac fibroblasts (Figure 2A) and observed that GLS1 expression was also significantly upregulated in P2 versus P0 cells, demonstrating that GLS1 expression increases as cardiac fibroblasts become myofibroblasts, whether by TGFβ_1_ treatment or passaging (Figure 2B).

Given the upregulation of GLS1 by TGFβ (Figure 1), we assessed the impact of scleraxis overexpression on the expression of various enzymes in the glutaminolysis pathway. Scleraxis was sufficient to induce dramatic upregulation of GLS1 mRNA expression by more than 20 fold (Figure 2C). Scleraxis also induced a tripling of GLS2 expression (Figure 2D), a doubling of GOT2 expression (Figure 2E), and a 70% increase in the expression of GDH1 (Figure 2F). Scleraxis significantly increased GLS1 protein expression by more than four-fold while having no impact on ALDOC expression (Figure 2G). Scleraxis thus impacts the expression of enzymes throughout the glutaminolysis pathway. Conversely, scleraxis overexpression significantly reduced the expression of Acot2 by approximately 50% (Figure 2G).

### 3.3. Scleraxis Is Critical for TGFβ-Mediated GLS1 Expression

**Figure 3:**
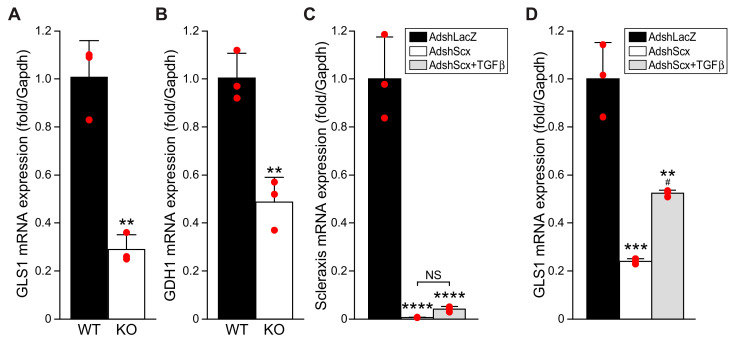
Loss of scleraxis attenuates glutaminolysis gene expression. (**A**,**B**) Cardiac fibroblasts were isolated from wild-type (WT) or scleraxis knockout (KO) mice and assayed for the expression of GLS1 (**A**) and GDH1 (**B**) by qPCR. (**C**,**D**) P1 rat cardiac fibroblasts were transfected with adenovirus encoding small hairpin RNA targeting LacZ (AdshLacZ) or scleraxis (AdshScx) for 72 h, with or without 10 ng/mL TGFβ_1_ addition after 48 h, and assayed by qPCR for expression of scleraxis (**C**) and GLS1 (**D**). Statistical analysis was by the two-tailed Student’s t-test (**A**,**B**) or by one-way ANOVA with the Tukey post hoc test (**C**,**D**) (n = 3). ** *p* < 0.01, *** *p* < 0.001, **** *p* < 0.0001 versus WT, or AdshLacZ; # *p* < 0.05 versus AdshScx. NS, not significant.

Given that scleraxis significantly upregulates the expression of multiple glutaminolysis enzymes (Figure 2), we assessed the requirement for scleraxis in the expression of key targets in this pathway. The expression of both GLS1 (Figure 3A) and GDH1 (Figure 3B) mRNA was significantly decreased in cardiac fibroblasts isolated from germline scleraxis knockout mice compared to wild-type animals. Similarly, the knockdown of scleraxis in P1 cardiac fibroblasts (Figure 3C) caused a downregulation of GLS1 expression by ~75% (Figure 3D). Notably, while TGFβ increased GLS1 expression following the knockdown of scleraxis (Figure 3D), GLS1 expression remained significantly below control cells, and the magnitude of the effect was dramatically lower than the upregulation of GLS1 observed following TGFβ treatment in scleraxis-intact cells (Figure 1E). Scleraxis thus appears to be both sufficient and necessary for normal GLS1 expression and induction by TGFβ.

### 3.4. Scleraxis Is Required for TGFβ-Induced Glutaminolysis Flux

**Figure 4:**
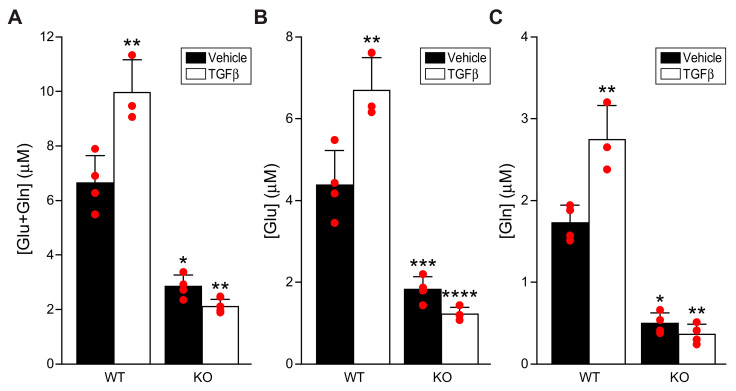
Loss of scleraxis attenuates TGFβ-induced glutaminolysis. (**A**–**C**) Cardiac fibroblasts were isolated from wild-type (WT) or scleraxis knockout (KO) mice, treated with vehicle or TGFβ_1_ (10 ng/mL) for 24 h, and assayed for intracellular [glutamate + glutamine] (**A**), [glutamate] (**B**), and [glutamine] (**C**). Statistical analysis was by two-way ANOVA with the Tukey post hoc test (n = 3–4). * *p* < 0.05, ** *p* < 0.01, *** *p* < 0.001, **** *p* < 0.0001 versus WT + Vehicle.

To determine the impact of TGFβ and scleraxis on glutaminolysis flux in P1 cardiac fibroblasts, we assayed cellular levels of glutamate and glutamine. In cells isolated from wild-type mice, TGFβ significantly increased levels of intracellular [glutamate + glutamine], indicative of greater glutaminolysis following TGFβ treatment (Figure 4A). Both intracellular [glutamate] and [glutamine] showed the same pattern, confirming that net flux through this pathway was increased (Figure 4B,C), rather than an increase solely in input or output. Notably, baseline cellular [glutamate + glutamine], [glutamate], and [glutamine] were significantly decreased in scleraxis knockout cells (Figure 4A–C). Furthermore, TGFβ completely failed to induce increased glutaminolysis in knockout cells. These results are congruent with the observed changes in glutaminolysis gene expression following scleraxis knockdown or knockout (Figure 3).

### 3.5. Scleraxis Transactivates the Human GLS1 Gene Promoter

**Figure 5:**
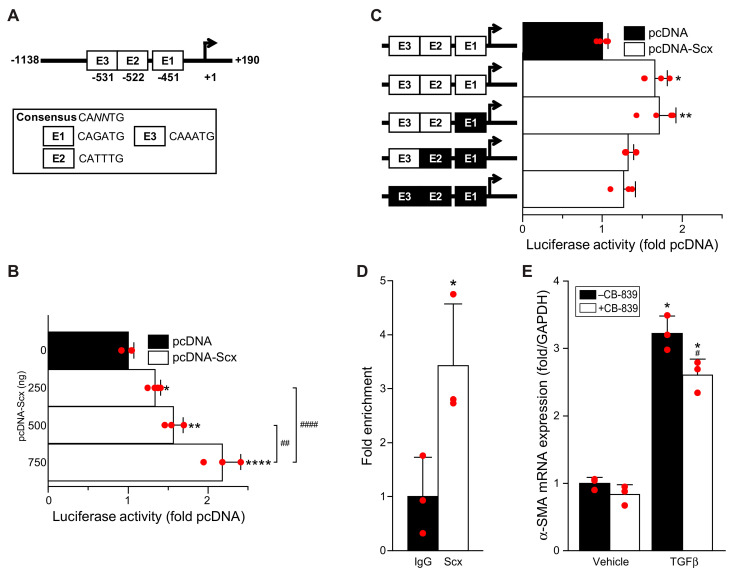
Scleraxis transactivates the human GLS1 gene promoter. (**A**) Schematic depicting the proximal ~1.1 kilobase human GLS1 gene promoter containing three putative E-box sequences (E1–E3); numbers depict distance in nucleotides relative to the transcription start site. (**B**) NIH3T3 fibroblasts were transfected with an hGLS1 promoter luciferase reporter vector plus empty vector (pcDNA) or plus vector encoding scleraxis (pcDNA-Scx) and assayed for luciferase expression. (**C**) NIH3T3 fibroblasts were transfected as in (**B**), with the hGLS1 promoter with either intact (white fill) or sequentially mutated (black fill) E-boxes, and assayed for luciferase activity. (**D**) Chromatin immunoprecipitation was performed in human adult cardiac myofibroblasts using antibodies to scleraxis (Scx) or IgG control and primers spanning the region encompassing E1 to E3, with amplification carried out by qPCR. (**E**) Human cardiac myofibroblasts were treated with 10 ng/mL TGFβ and/or 0.3 μΜ CB-839 for 24 h, then assayed for α-SMA expression by qPCR. Statistical analysis was by one-way ANOVA with the Tukey post hoc test (**B**) (n = 3–4), or by the Kruskal–Wallis test followed by Dunn’s multiple comparisons test (**C**) (n = 3–4), or by the two-sided Student’s t-test (**D**) (n = 3), or by two-way ANOVA with Tukey (**E**) (n = 3). * *p* < 0.05, ** *p* < 0.01, **** *p* < 0.0001 versus pcDNA or versus IgG or versus vehicle; # *p* < 0.05, ## *p* < 0.01, #### *p* < 0.0001 versus samples as indicated (**B**) or versus TGFβ alone (**E**).

While scleraxis gain- or loss-of-function altered the expression of various glutaminolysis pathway enzymes, the most potent impact of scleraxis was on the expression of the rate-limiting enzyme GLS1. We therefore examined whether scleraxis transactivates the human GLS1 gene promoter. The proximal ~1.1 kilobase promoter contains three putative E-boxes—consensus sequences of CA*NN*TG to which scleraxis may bind (Figure 5A) [24]. Scleraxis transactivates similar E-box sequences in various pro-fibrotic gene promoters, including Col1α2, αSMA, fibronectin, and MMP2 [5,17,18,19,20].

The proximal hGLS1 promoter sequence was cloned into luciferase expression vector pGL4.10 for luciferase reporter assays in NIH3T3 mouse embryonic fibroblasts. Scleraxis induced a dose-dependent, stepwise increase in reporter transactivation (Figure 5B). To identify which putative E-boxes are most critical for scleraxis-mediated hGLS1 promoter transactivation, site-directed mutagenesis of E-boxes 1, 2, and 3 was used to attenuate scleraxis binding. The mutation of E-box 1 alone had no impact on hGLS1 transactivation by scleraxis (Figure 5C). In contrast, the mutation of E-box 2 in conjunction with E-box 1 reduced transactivation, while the additional mutation of E-box 3 had no further effect. These results indicate that E-box 2 is most critical for scleraxis-mediated transactivation of the hGLS1 promoter.

The binding of scleraxis to the human GLS1 promoter was validated by chromatin immunoprecipitation (ChIP) assay in human cardiac myofibroblasts. While ChIP is unable to distinguish binding to any specific E-box given their close spacing in this promoter (80 base pairs between E1 and E3), scleraxis binding was significantly enriched in this region compared to non-specific IgG (Figure 5D). The upregulation of α-smooth muscle actin (α-SMA) mRNA by TGFβ was significantly reduced by CB-839 in these cells (Figure 5E), suggesting that the role of glutaminolysis in fibroblast activation identified in rat and mouse cells (Figure 1, Figure 2, Figure 3 and Figure 4) is similarly important in human cells.

## 4. Discussion

Interstitial fibroblasts normally synthesize the extracellular matrix components of the healthy heart [25,26]. During wound healing or in response to stress, however, it is the myofibroblasts that play a major role through the accelerated synthesis of extracellular matrix components such as fibrillar collagens. Persistent fibrosis contributes to structural and functional remodeling of the heart, with increased arrhythmogenesis due to altered conduction characteristics, leading to further functional and structural derangements that may cause heart failure and death [1,8]. At present, cardiac fibrosis remains without existing interventions for clinical management.

**Figure 6:**
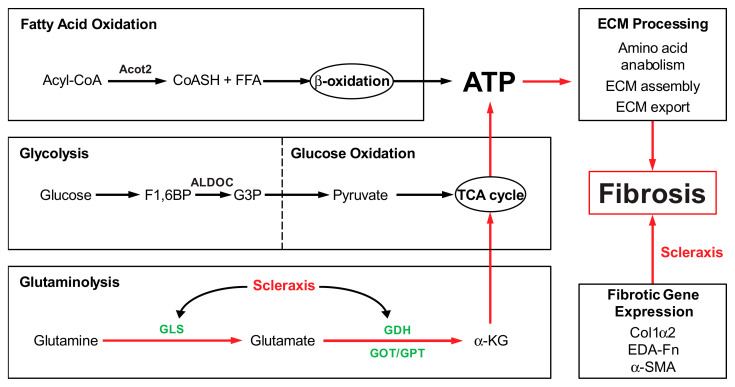
Scleraxis (red text) regulation of gene expression in fibrosis. Extracellular matrix (ECM) synthesis and processing require large quantities of ATP, which may be derived from various intracellular metabolic pathways, including fatty acid b-oxidation, glycolysis, glucose oxidation, and glutaminolysis. Key enzymes of each pathway are depicted, including those of glutaminolysis (green text). Red arrows highlight mechanisms that may contribute to cardiac fibrosis: scleraxis regulates GLS1 expression, per the present results, and shows evidence of regulation of other glutaminolysis enzyme genes. Scleraxis also directly transactivates a variety of pro-fibrotic genes, including ECM components Col1α2 and EDA-Fn; thus, scleraxis may regulate fibrosis by controlling both ECM gene expression and glutaminolysis genes to provide the energy necessary to support fibrosis.

Despite the significant impact of fibrosis on cardiac function and its key role in patient morbidity and mortality, surprisingly little is known of the energy metabolism of cardiac fibroblasts and myofibroblasts, although some advances have been made in other tissues. Increased glycolysis enhances myofibroblast activation and contributes to lung fibrosis [8,9]. Recent studies in mouse embryonic fibroblasts showed that fibrotic signaling increased α-KG availability, with increases in glycolysis and glutaminolysis to provide TCA cycle intermediates [15]. Glutaminolysis has also been identified as a key energy source for liver and lung fibrosis [13,14]. Our results suggest that a similar requirement for glutaminolysis occurs when cardiac fibroblasts are activated to become myofibroblasts (Figure 6).

TGFβ is a potent driver of fibroblast activation to myofibroblasts, contributing to fibrosis in many tissue types, and we observed that TGFβ increased the expression and secretion of the myofibroblast-specific marker periostin, increased stress fiber formation, and induced a transient increase in cell proliferation concomitant with a dramatic increase in GLS1 expression. Intriguingly, all of these effects could be attenuated by the GLS1 inhibitor CB-839, suggesting that the blockade of glutaminolysis prevented fibroblast activation and metabolic reprogramming. Although we did not assess fatty acid β-oxidation, it is notable that CB-839 also restored the loss of Acot2 expression induced by TGFβ, suggesting that an increase in glutaminolysis may be accompanied by a decrease in this metabolic pathway. While increased glycolysis has been implicated in lung fibroblast activation, we observed no change in ALDOC expression by either TGFβ or CB-839, suggesting the cell type specificity of this pathway. Passaging fibroblasts to induce activation to myofibroblasts, or overexpressing scleraxis to induce activation, caused similar increases in GLS1 expression, and scleraxis also increased the expression of other glutaminolysis pathway components, including GLS2, GOT2, and GDH1. The activation of cardiac fibroblasts to myofibroblasts, regardless of the inducer, thus increases gene expression indicative of an increase in glutaminolysis. We also observed increases in intracellular glutamate and glutamine, indicative of greater flux through the glutaminolysis pathway, in response to TGFβ. Together, these findings support a central role for the induction of glutaminolysis during cardiac fibroblast activation, similar to observations in the liver and lung.

TGFβ potently induces downstream pro-fibrotic gene programs via both the canonical Smad pathway and a variety of non-canonical signal transduction mechanisms. We have previously shown that TGFβ induces scleraxis expression via both mechanisms, i.e., Smad3 and Erk1/2 [27]. We have also reported that scleraxis not only induces the expression of myriad pro-fibrotic genes via direct promoter transactivation, but also is critically required for TGFβ to regulate the expression of these genes, ostensibly via the physical interaction of scleraxis with Smad3 [5,17,18,19,20]. Our data suggest that a similar mechanism is involved in the induction of glutaminolysis, since the TGFβ-mediated upregulation of GLS1 or increases in intracellular glutamine and glutamate required scleraxis. As a basic helix-loop-helix transcription factor, scleraxis regulates gene expression by binding to E-box sequences within target gene promoters, including fibrillar collagens, fibronectin, MMP2, αSMA, Twist1, Snai1, and vimentin [5,17,18,19,20,28]. Luciferase assays confirm that, while the proximal human GLS1 promoter contains three sites that conform to the E-box consensus sequence CA*NN*TG, only one of these sites (E2) appears to be critical for scleraxis-mediated promoter transactivation. The control of GLS1 expression by scleraxis thus exhibits the same hallmarks of many other scleraxis target genes involved in fibroblast activation and ECM expression, suggesting that GLS1 upregulation (and increased glutaminolysis) may be part of this pro-fibrotic gene program. While we did not examine other glutaminolysis genes in the same detail as GLS1, our data suggest that scleraxis may similarly regulate the expression of GOT2 and GDH1, providing multiple sites of control over glutaminolysis.

Given its central role in patient morbidity and mortality, interfering with fibrosis is a critical, unmet clinical need in many tissue types, including the heart. The present work suggests two approaches that may help to address this gap—interfering with scleraxis to attenuate GLS1 expression or blocking GLS1 function by pharmacologic inhibition. Removing glutaminolysis as a key source of cellular energy has already been demonstrated to be effective in cancer therapy [29,30]. GLS1 is abnormally upregulated in a variety of cancers, and depleting or pharmacologically inhibiting GLS1 with CB-839 reduced tumour proliferation and metastasis [31,32,33]. CB-839 has also been effective in reducing pulmonary or hepatic fibrosis [13,14]. Our data suggest that this approach could be similarly effective in the heart. Knocking down scleraxis dramatically reduced GLS1 expression, and scleraxis knockout cells failed to increase glutamine and glutamate levels in response to TGFβ. CB-839 blocked the TGFβ-mediated upregulation of both GLS1 and periostin, suggesting a blockade of fibroblast activation. An important next step will be to determine whether CB-839 administration in vivo attenuates cardiac fibrosis in preclinical models.

## 5. Conclusions

Our study reveals that GLS1 plays an important role in the activation of cardiac fibroblasts to myofibroblasts and that the TGFβ-mediated upregulation of GLS1 is dependent on scleraxis, which directly transactivates the human GLS1 gene promoter. Glutaminolysis thus appears to be important in cardiac fibroblast activation, similar to prior observations in the liver and lung, suggesting a common mechanism of ramping up energy production in response to increased demands during tissue fibrosis. Targeting scleraxis and/or GLS1 may thus be effective in attenuating the energy supply in order to starve the fibrotic process.

## Supplementary Materials

The following supporting information can be downloaded at: https://www.mdpi.com/article/10.3390/cells11091471/s1, Table S1: List of qPCR primers. Table S2: List of primers for cloning the hGLS1 promoter. Table S3: List of primers for mutagenesis of hGLS1 promoter E-boxes. Table S4: List of primers for chromatin immunoprecipitation.
