# Supplementary material for: Regulation of Cardiac Fibroblast GLS1 Expression by Scleraxis
Source: Cells. 2022 Apr 27;11(9):1471. doi: 10.3390/cells11091471 (PMC9101234; doi:10.3390/cells11091471)
Supplement: Supplementary file 1 [file cells-11-01471-s001.zip › cells-1637252-supplementary.pdf]

Supplementary Materials

Table S1. List of qPCR primers. Forward (F) and reverse (R) primers are indicated, yielding amplicons of the indicated size.

Primer	Sequence	Size (bp)
Scx-F (rat/mouse)	5'-ACAGATCTGCACCTTCTG-3'	177
Scx-R (rat/mouse)	5'-GCTCAGATCAGGTCCAA-3'	
GLS1-F (rat/mouse)	5'-GCAAACCTTCGGAGGGCAGACA-3'	378
GLS1-R (rat/mouse)	5'-GCTCAGTCCTGAGGCCGTTCG-3'	
Periostin-F (rat)	5'-TCGTGGAACCAAAAATTAAAGTC-3'	77
Periostin-R (rat)	5'-CTTCGTCATTGCAGGTCCTT-3'	
ACTA2-F (human)	5'-CTATGCCTCTGGACGCACAACT-3'	115
ACTA2-R (human)	5'-CAGATCCAGACGCATGATGGCA-3'	
Aldolase C-F (rat/mouse)	5'-AACACTGAGGAGAATCGCCG-3'	172
Aldolase C-R (rat/mouse)	5'-TGATGCCTACGAGAATGCCC-3'	
Acot2-F (rat/mouse)	5'-CCTTGCTATGGCCTCCTTCC-3'	178
Acot2-R (rat/mouse)	5'-TTGCAGAGCTTCCACGACAT-3'	
GLS2-F (rat)	5'-TCCACATGGCCTGTGAACTCCTCA-3'	300
GLS2-R (rat)	5'-CAGTGGCATGTTGCCTCGACTTG-3'	
GDH1-F (rat/mouse)	5'-ATCAATTCTGGGCTTCCCCAA-3'	116
GDH1-R (rat/mouse)	5'-TGGGTGCATTGGATTTGGTC-3'	
GOT2-F (rat)	5'-CCAAGACTTGCGGCTTTGAC-3'	152
GOT2-R (rat)	5'-CTTTTTCTTCACCACCGCCG-3'	
Gapdh-F (mouse)	5'-TCACCACCATGGAGAAGGC-3'	169
Gapdh-R (mouse)	5'-GCTAAGCAGTTGGTGGTGCA-3'	
Gapdh-F (rat)	5'-GCAAGTTCAACGGCACAG-3'	140
Gapdh-R (rat)	5'-GCCAGTAGACTCCACGACAT-3'	
GAPDH-F (human)	5'-GTCTCCTCTGACTTCAACAGCG-3'	131
GAPDH-R (human)	5'-ACCACCCTGTTGCTGTAGCCAA-3'	

Table S2. List of primers for cloning the hGLS1 promoter. Cloned restriction sites for SacI (forward primer) and EcoRV (reverse primer) are indicated in boldface.

Forward primer	5'-ACGT**GAGCTC**CCTGGGCGACAGAGT-3'
Reverse primer	5'-GGCC**GATATC**CGCGGTCTGTGGTGG-3'

Table S3. List of primers for mutagenesis of hGLS1 promoter E-boxes. E-box sequences are in boldface; mutated nucleotides are underlined.

ΔE1-GLS1-F	5'-CCCTGACCTGGGGGC**CTAAAG**CGAGCTGGGATCGGC-3'
ΔE1-GLS1-R	5'-GCCGATCCCAGCTCG**CTTTAG**GCCCCCAGGTCAGGG-3'
ΔE2-GLS1-F	5’-TTCAAG**CAAATG**TTA**CTTTAG**GGAGAGGGATGAAGC-3’
ΔE2-GLS1-R	5'-GCTTCATCCCTCTCC**CTAAAG**TAA**CATTTG**CTTGAA-3'
ΔE2,3-GLS1-F	5'-ATGGAAAGTTTCAAG**CTAACG**TTA**CTTTAG**GGAGAG-3'
ΔE2,3-GLS1-R	5'-CTCTCC**CTAAAG**TAA**CGTTAG**CTTGAAACTTTCCAT-3'

Table S4. List of primers for chromatin immunoprecipitation. Forward (F) and reverse (R) primers are indicated.

hGLS1-ChIP-F	5'-ACTTCACGTCTGGGTAGTAGC-3'
hGLS1-ChIP-R	5'-GTCGGAACTCAGCCGATCC-3'
